# New Appliances and Things Medical

**Published:** 1904-03-19

**Authors:** 


### NEW APPLIANCES AND THINGS MEDICAL.

### HUMANISED MILK TABLETS.

There is little doubt that the ideal food for infants whose mothers will not or cannot nurse them is cow's milk suitably modified. But there remains the practical difficulty that the methods suggested for "humanising" cow's milk are usually either too complicated to be easily carried out at home or else are imperfect in their results. To obviate these drawbacks, the "Humanised Milk Tablets" have been placed on the market. Two sets of tablets are supplied—large ones of lactose and smaller ones containing rennet together with some sodium bicarbonate and calcium phosphate. The *rationale* of using these preparations is set forth in detail in an article which appeared in The Hospital of January 9th, 1904. Briefly stated it is as follows:—A pint of cow's milk is divided into two equal parts. One half is skimmed, and the skim is added to the other half. The skimmed portion is heated to 100° F., and one large and one small tablet, previously crushed, are added, and the milk kept at about the same temperature for 20 minutes. The curd is then broken up, and the whey strained off and added to the second half-pint of cow's milk, with an extra six tablespoonfuls of fresh cow's milk added. The whole may then be sterilised.

We have tested this method and find it works admirably, while it is so easy that it can readily become a matter of domestic routine.

The tablets, with directions for using them, may be obtained from Mr. A. G. Felthouse, M.P.S., 30 Highbury Park, London, N.

### ANGIER'S PETROLEUM EMULSION.

(The Angier Chemical Company, Limited, 31 and 32 Snow Hill, London, E.C.)

This preparation is now so thoroughly established as a valuable therapeutic agent, that every active practitioner is well acquainted with its uses. The emulsion contains to each ounce 33½ per cent. of pure petroleum and nine grains of the sodium and calcium hypophosphites. The petroleum, which is obtained from the best American wells, has been rendered tasteless by freeing it from the organic acids and sulphur compounds which give to the crude oil its unpleasant taste and odour. Although petroleum is practically inert chemically, and is excreted unchanged from the body, it possesses certain important properties. It is an ideal demulcent, having no tendency to decompose and form irritating acids, while it has been demonstrated that petroleum emulsion hastens the process of the digestion of albumen. The addition of calcium and sodium hypophosphites, which act as nutritive tonics, is highly appropriate. Thus the preparation is especially useful in cases of malnutrition with digestive disturbance, and in irritable conditions of the upper respiratory tract.

### THE "WALL" AUTOMATIC SASH LOCK.

(R. Clinton Hughes, 56 Gracechurch Street, London, E.C.)

By means of this ingenious contrivance it is possible to have a window open and yet securely locked. Its scope of usefulness is therefore a wide one. Not only is the householder able to admit a plentiful supply of fresh air without the fear of having his house burgled, but various institutions also may find a ready protection against the escape of irresponsible inmates. The apparatus is fixed to the upper surface of the lower window sash. It possesses a spring bolt which shoots into holes prepared for it in the upper sash. If desired a padlock may be used, so that, once fixed in place, there is no chance of evilly-intentioned persons unbolting the window. With the lock fixed it is possible to have either the upper or the lower half of the window open, or both halves simultaneously. There is one more advantage which may be claimed for this invention, and that is that it obviates the annoying rattling of windows during boisterous weather. The contrivance is small and inoffensive to the eye. It is made in different sizes, and when ordering it is necessary to mention the thickness of the lower window sash. The price is 1s. 6d. each.

